# Filamentous aggregations of phosphorylated α-synuclein in Schwann cells (Schwann cell cytoplasmic inclusions) in multiple system atrophy

**DOI:** 10.1186/s40478-015-0208-0

**Published:** 2015-05-21

**Authors:** Keiko Nakamura, Fumiaki Mori, Tomoya Kon, Kunikazu Tanji, Yasuo Miki, Masahiko Tomiyama, Hidekachi Kurotaki, Yasuko Toyoshima, Akiyoshi Kakita, Hitoshi Takahashi, Masahito Yamada, Koichi Wakabayashi

**Affiliations:** Department of Neuropathology, Institute of Brain Science, Hirosaki University Graduate School of Medicine, 5 Zaifu-cho, Hirosaki, 036-8562 Japan; Department of Neurology and Neurobiology of Ageing, Kanazawa University Graduate School of Medical Science, Kanazawa, Japan; Department of Neurology, Aomori Prefectural Central Hospital, Aomori, Japan; Department of Pathology, Aomori Prefectural Central Hospital, Aomori, Japan; Department of Pathology, Brain Research Institute, University of Niigata, Niigata, Japan; Department of Pathological Neuroscience, Center for Bioresource-based Researches, Brain Research Institute, University of Niigata, Niigata, Japan

**Keywords:** α-synuclein, Multiple system atrophy, Peripheral nerve, Schwann cell, Schwann cell cytoplasmic inclusion, Ultrastructure

## Abstract

**Background:**

The histological hallmark of multiple system atrophy (MSA) is the presence of filamentous aggregations of phosphorylated α-synuclein in oligodendrocytes, referred to as glial cytoplasmic inclusions (GCIs). Although GCIs can occur widely in the central nervous system, accumulation of phosphorylated α-synuclein in Schwann cells has not been reported in MSA. We immunohistochemically examined the cranial and spinal nerves, peripheral ganglia and visceral autonomic nervous system of patients with MSA (n = 14) and control subjects (n = 20).

**Results:**

In MSA, accumulation of phosphorylated α-synuclein was found in the cytoplasm of Schwann cells. These Schwann cell cytoplasmic inclusions (SCCIs) were also immunopositive for ubiquitin and p62. SCCIs were found in 12 of 14 patients with MSA (85.7 %). They were most frequent in the anterior nerve of the sacral cord and, to a lesser extent, in the cranial nerves (oculomotor, glossopharyngeal-vagus and hypoglossal nerves), and spinal and sympathetic ganglia. SCCIs were rarely found in the visceral organs. Immunoelectron microscopy demonstrated that the SCCIs consisted of abnormal filaments, 15–20 nm in diameter. No such inclusions were found in controls.

**Conclusion:**

The present findings indicate that Schwann cells are also involved in the disease process of MSA.

## Introduction

Multiple system atrophy (MSA) is an adult-onset neurodegenerative disorder manifested clinically as a combination of parkinsonism, cerebellar ataxia and autonomic dysfunction. MSA is now divided into two clinical subtypes: MSA with predominant parkinsonian features (MSA-P) and MSA with predominant cerebellar dysfunction (MSA-C) [1]. MSA is characterized pathologically by any combination of coexisting olivopontocerebellar atrophy, striatonigral degeneration and preganglionic autonomic lesions [2]. The histological hallmark of MSA is widespread glial cytoplasmic inclusions (GCIs) in the central nervous system [3–6]. These GCIs can be visualized by silver staining such as the Gallyas-Braak method [3], and ultrastructurally they consist of granule-associated filaments 20–30 nm in diameter [3, 4, 7]. The major component of GCIs is α-synuclein [8], which is phosphorylated at Serine 129 [9] and ubiquitinated [10]. Although primary oligodendroglial pathology is the main feature of MSA [11–13], accumulation of phosphorylated α-synuclein is also consistently found in the neuronal cytoplasm, processes and nuclei [14]. Similar neuronal inclusions are found less frequently in the peripheral sympathetic ganglia [13, 15].

Although immunoreactivity of non-phosphorylated α-synuclein has been reported in normal and neoplastic Schwann cells in the peripheral nervous system of humans [16], accumulation of phosphorylated α-synuclein in Schwann cells of patients with MSA has not been described hitherto. Here we immunohistochemically examined the cranial and spinal nerves, peripheral ganglia and visceral autonomic nervous system of patients with MSA using antibodies against phosphorylated α-synuclein, and report for the first time that Schwann cells in these patients are also affected by filamentous aggregations of phosphorylated α-synuclein.

## Materials and methods

### Subjects

Thirty-four autopsy cases were included in this study. Fourteen of the patients (age 49–79 years, average = 64.6 years) had a clinical history of MSA, which was confirmed at autopsy by the presence of numerous GCIs (Table 1). All of the MSA cases lacked Lewy body pathology. The clinical and neuropathological features of early MSA (cases 2 and 12) have been reported previously [17, 18]. Twenty patients used as controls (age 40–84 years, average = 70.0 years) were clinically and histopathologically free of neurodegenerative disease. This study was approved by the Institutional Ethics Committee of Hirosaki University Graduate School of Medicine.

**Table 1:** Summary of clinical findings of patients with multiple system atrophy (MSA)

Case No.	Age at death (years)	Gender	Disease duration (years)	Clinical diagnosis	Pathological diagnosis	Initial symptoms	Symptoms						
							Parkinsonian signs	Cerebellar signs	OH	UD	Constipation	Dyshidrosis	Impotence
1	49	F	7	MSA-P	MSA-P	limping	+	−	−	+	+	+	−
2	57	F	1	spinocerebellar degeneration	MSA-C	ataxia	−	+	−	−	−	−	−
3	58	M	7	SDS	MSA-C	UD, constipation, impotence	+	+	+	+	+	−	+
4	61	M	7	MSA-P	MSA-P	UD	+	+	−	+	+	−	−
5	61	F	4.5	MSA-P	MSA-P	tremor	+	+	−	+	+	−	−
6	63	F	3	MSA-P	MSA-P	gait disturbance	+	−	+	+	−	−	−
7	65	M	7	MSA-C	MSA-C	gait disturbance	+	+	−	+	+	−	−
8	65	M	14	MSA-C	MSA-P	gait disturbance	+	+	+	+	−	−	−
9	66	M	9	SDS	MSA-P	UD	+	+	+	+	+	−	−
10	66	M	13	MSA-C	MSA-C	sensory disturbance	+	+	+	+	+	−	−
11	69	M	8	SDS	MSA-C	snoring	−	+	+	+	+	−	−
12	71	F	<1	neurologically normal	MSA-C	none	−	−	−	−	−	−	−
13	75	M	8	progressive supranuclear palsy	MSA-P	gait disturbance	+	+	+	+	−	−	−
14	79	M	4	SDS	MSA-C	unsteady gait	−	+	+	+	−	−	−

OH, orthostatic hypotension; UD, urogenital dysfunction; SDS, Shy-Drager syndrome; +, present;−, absent

### Immunohistochemistry

Immunohistochemical analysis was carried out using formalin-fixed, paraffin-embedded, 4-μm-thick sections from the midbrain, upper pons, medulla oblongata, spinal cord (cervical, thoracic, lumbar and sacral segments), and dorsal root and paravertebral sympathetic ganglia. Oculomotor and trigeminal nerves were examined at the level of the midbrain and upper pons, respectively. Glossopharyngeal and vagus nerves were examined at the level of the dorsal vagal nucleus. Since it was difficult to differentiate glossopharyngeal nerve from vagus nerve on the sections, these two nerves were described as a whole. Hypoglossal nerves were examined at the level of the gracile nucleus. Paraffin sections were also cut from block samples of the esophagus, stomach, small intestine, colon, heart, lung, thyroid, liver, pancreas, kidney, adrenal gland and urinary bladder. The sections were subjected to immunohistochemical processing using the avidin-biotin-peroxidase complex method with diaminobenzidine as the chromogen. The primary antibodies used were mouse monoclonal antibodies against phosphorylated α-synuclein (#64; Wako, Osaka, Japan; 1:5,000), aggregated α-synuclein (5G4; EMD Millipore, Temecula, CA, USA; 1:1,000) [19] and ubiquitin (1B3; MBL, Nagoya, Japan; 1:2,000), rabbit monoclonal antibody against phosphorylated α-synuclein (EP1536Y; Abcam, Cambridge, UK; 1:5,000), and rabbit polyclonal antibody against p62 (MBL; 1:1,000). #64 is a monoclonal antibody against a synthetic peptide corresponding to amino acid residues 124–134 of human α-synuclein with a phosphorylated Serine 129 residue. EP1536Y is also a monoclonal antibody against a synthetic peptide corresponding to residues surrounding phosphorylated Serine 129 of human α-synuclein.

In addition to routine immunohistochemical techniques, selected sections from the spinal cord of MSA patients were first stained using the modified Gallyas-Braak method [20]. The spinal nerve roots were observed under a ×40 objective lens. After removing the cover glasses from the slides using xylene, the specimens were decolorized in alcohol, then immunostained with anti-phosphorylated α-synuclein (Wako; 1:5,000). The spinal nerve roots were then observed again under a ×40 objective lens.

Semiquantitative assessment of inclusions in Schwann cells was performed in each region by anti-phosphorylated α-synuclein immunolabeling. The numbers of inclusions were estimated as: −, none; +, 1 to 5 inclusions; ++, >5 inclusions.

### Double immunostaining

To characterize the inclusion-bearing cells, anti-S-100 was used as a marker of Schwann cells [21], anti-tubulin polymerization promoting protein (TPPP)/p25α as a marker of oligodendroglia [22], and anti-phosphorylated neurofilament as a marker of axons [23]. TPPP/p25α is also known to be a component of GCIs in MSA [24]. Double immunofluorescence analysis was also performed to detect overlapping expression of phosphorylated α-synuclein and ubiquitin. Paraffin sections from the spinal cord of patients with MSA (n = 3) were processed for double-label immunofluorescence. Deparaffinized sections were blocked with donkey serum and then incubated overnight at 4 °C with a mixture of the monoclonal anti-phosphorylated α-synuclein (Wako; 1:500) and polyclonal anti-S-100 (DAKO, Tokyo, Japan; 1:500), anti-TPPP/p25α (Sigma-Aldrich Japan, Tokyo, Japan; 1:500) or anti-ubiquitin (DAKO; 1:200), or a mixture of the mouse monoclonal anti-phosphorylated neurofilament (SMI31; Cosmo Bio, Tokyo, Japan; 1:500) and rabbit monoclonal anti-phosphorylated α-synuclein (Abcam; 1:500). The sections were then rinsed and incubated for 1 h at 38 °C with anti-rabbit IgG tagged with Alexa Fluor 488 (Invitrogen, Carlsbad, CA, USA; 1:200) and anti-mouse IgG tagged with Alexa Fluor 594 (Invitrogen; 1:200), or anti-rabbit IgG tagged with Alexa Fluor 594 (Invitrogen; 1:200) and anti-mouse IgG tagged with Alexa Fluor 488 (Invitrogen; 1:200). The sections were examined using an Olympus BX63 fluorescence microscope (Olympus, Tokyo, Japan).

### Immunoelectron microscopy

The anterior spinal nerve roots from a case of MSA (case 1) were processed for immunoelectron microscopy. Fifty-micrometer-thick vibratome sections were cut from the formalin-fixed tissue. The sections were incubated with a rabbit monoclonal anti-phosphorylated α-synuclein antibody (Abcam; 1:500), followed by incubation with a biotinylated secondary anti-rabbit IgG (Vector, Burlingame, CA, USA; 1:200) and avidin-biotin-peroxidase complex (Vector; 1:200), and the reaction was developed with diaminobenzidine. The immunolabeled sections were post-fixed in 1 % glutaraldehyde and 1 % osmium tetroxide, dehydrated in ethanol, and then embedded in Poly/Bed 812 resin (Polysciences, Inc., Warrington, PA, USA). Ultrathin sections were cut and viewed with a JEOL1230 electron microscope (JEOL Ltd., Tokyo, Japan).

## Results

### Morphology and immunohistochemical features

**Fig. 1:**
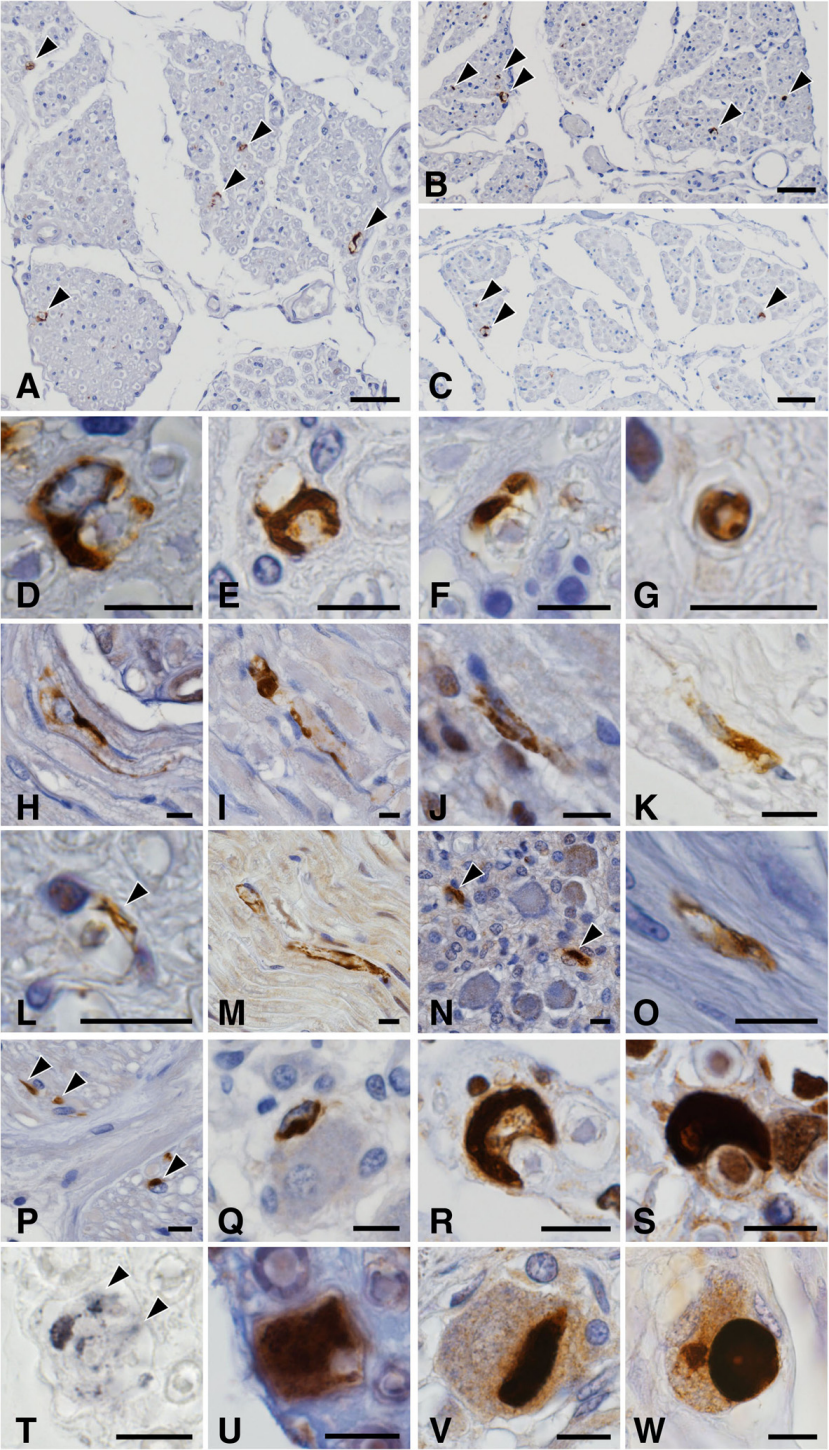
Schwann cell **(a–u)** and neuronal **(v**, **w)** cytoplasmic inclusions stained with anti-phosphorylated α-synuclein **(a**, **b**, **d**–**q**, **u–w)**, anti-aggregated α-synuclein **(c)**, anti-ubiquitin **(r)**, anti-p62 **(s)** and the Gallyas-Braak method **(t)**. **a–i** Schwann cell cytoplasmic inclusions (SCCIs) (arrowheads) in the anterior spinal nerve roots. SCCIs displaying crescent-shaped **(d)**, coil-like **(e)**, or cigar-shaped morphology **(f)**. SCCIs enwrapping the axons **(g)**. SCCIs extending their processes to the axons **(h**, **i)**. **j–l** SCCIs in the oculomotor **(j)**, glossopharyngeal-vagus **(k)** and hypoglossal **(l)** nerves. **m** and **n** SCCIs in the dorsal root **(m)** and sympathetic **(n)** ganglia. **o**–**q** SCCIs in the stomach **(o)**, adrenal grand **(p)** and urinary bladder **(q)**. **r** and **s** SCCIs showing immunopositivity for ubiquitin **(r)** and p62 (**s**). **t** and **u** Sequential staining of the same sections of the spinal nerve with Gallyas-Braak **(t)** and anti-phosphorylated α-synuclein **(u)**. SCCIs (arrowheads) are only weakly or partially stained with the Gallyas-Braak method. **v** and **w** Neuronal cytoplasmic inclusions in the dorsal root ganglia. Immunostaining with anti-phosphorylated α-synuclein antibodies (#64 for **a**, **e**, **g**–**q**, **u**–**w**; and EP1536Y for **b**, **d**, **f)**. Bars = 50 μm in **a**–**c**; 10 μm in **d**–**w**

Immunostaining with anti-phosphorylated and anti-aggregated α-synuclein antibodies as well as the modified Gallyas-Braak method demonstrated widespread occurrence of GCIs throughout the brain and spinal cord of patients with MSA, but not in control subjects. The immunostaining with two monoclonal anti-phosphorylated α-synuclein antibodies and a monoclonal anti-aggregated α-synuclein antibody revealed Schwann cell cytoplasmic inclusions (SCCIs) in the cranial and spinal nerves, peripheral ganglia and visceral autonomic nervous system of MSA patients (Fig. 1a–q). They appeared crescent-shaped, coil-like, or cigar-shaped (Fig. 1d–f). The SCCIs enveloped the axons (Fig. 1g) and extended their processes from the cytoplasm to the axons (Fig. 1h, i). Similar inclusions were detected with anti-ubiquitin and anti-p62 antibodies (Fig. 1r, s). The inclusions could not be visualized with hematoxylin and eosin, Klüver-Barrera or Bodian’s method. GCIs appeared argyrophilic with the modified Gallyas-Braak method, whereas SCCIs were stained only weakly or partially (Fig. 1 t, u). No such inclusions were found in controls.

**Fig. 2:**
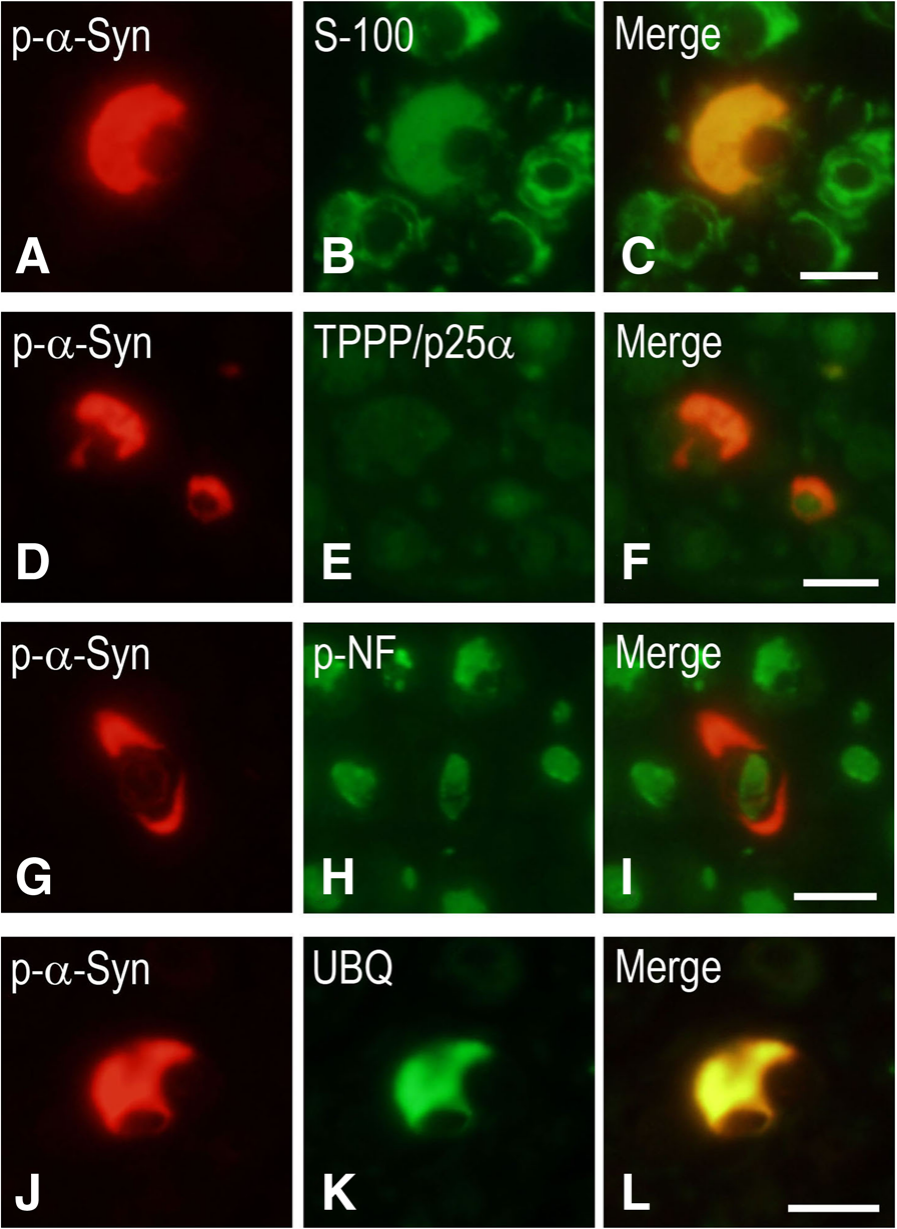
Double immunofluorescence staining of Schwann cell cytoplasmic inclusions. Co-localization of phosphorylated α-synuclein (p-α-Syn) and S-100 **(a–c)**, but not TPPP/p25α **(d–f)** or phosphorylated neurofilament (p-NF) **(g–i)**, in the inclusions. p-α-Syn and ubiquitin (UBQ) are also co-localized in the inclusions **(j–l)**. P-α-Syn **(a**, **d**, **g**, **j)** appears red, S-100 **(b)**, TPPP/p25α **(e)**, p-NF **(h)** and UBQ **(k)** appear green, and overlap of S-100 or UBQ and p-α-Syn **(c**, **l)** appears yellow. Bars = 10 μm

To further characterize the inclusion-bearing cells, anti-S-100 was used as a Schwann cell marker, anti-TPPP/p25α as an oligodendroglia marker, and phosphorylated neurofilament as an axon marker. Double immunofluorescence analysis revealed co-localization of phosphorylated α-synuclein and S-100 (Fig. 2a–c), but not TPPP/p25α (Fig. 2d–f) or phosphorylated neurofilament (Fig. 2g–i), in the inclusions. Phosphorylated α-synuclein and ubiquitin were also co-localized in the inclusions (Fig. 2 j–l).

### Immunoelectron microscopy

**Fig. 3:**
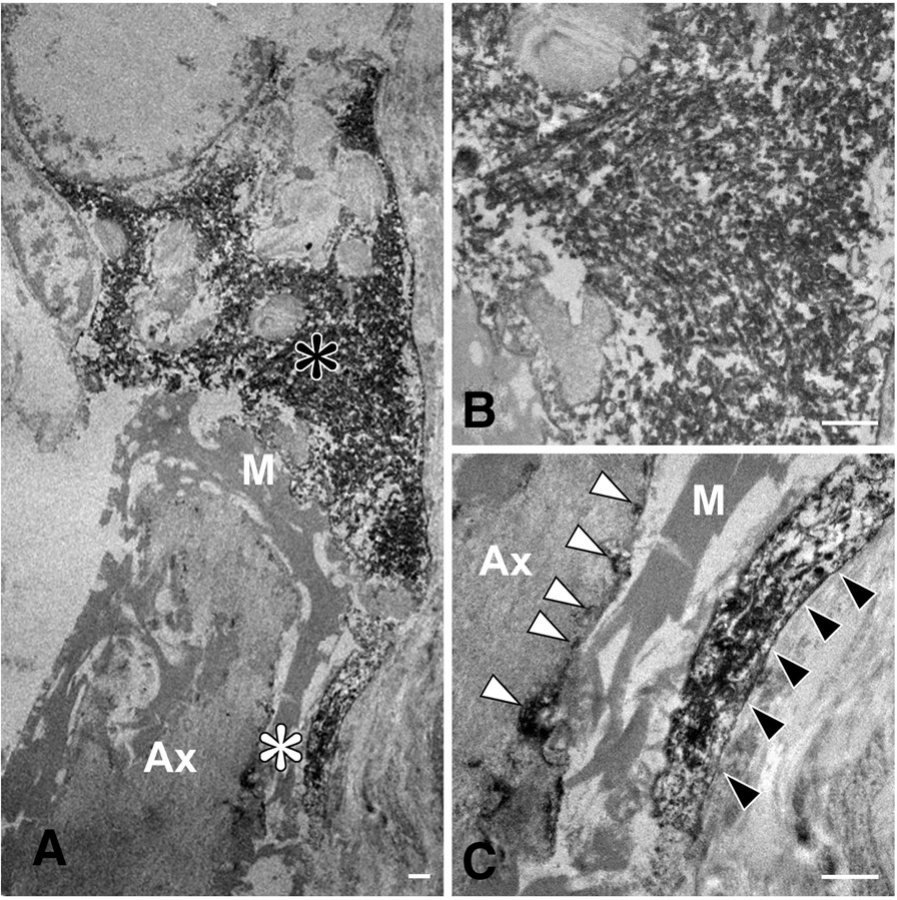
Immunoelectron microscopy of Schwann cell cytoplasmic inclusions in the spinal nerve roots. **a** Phosphorylated α-synuclein-immunoreactive structures in the cytoplasm of Schwann cells. **b** A higher-magnification view of the area indicated by the black asterisk in **(a)**. The inclusion showing granule-coated fibrillary structures, about 15–20 nm in diameter. Anti-phosphorylated α-synuclein antibody labels filamentous and granular structures. **c** A higher-magnification view of the area indicated by the white asterisk in **(a)**. Phosphorylated α-synuclein-immunoreactive structures are evident in the outer (black arrowheads) and inner loops (white arrowheads) of Schwann cells. M, myelin; Ax, axon. Bars = 1 μm

Pre-embedding immunoelectron microscopy demonstrated phosphorylated α-synuclein-immunoreactive structures in the cytoplasm of Schwann cells (Fig. 3a). The SCCIs consisted of randomly arranged, loosely packed, granule-coated fibrils, approximately 15–20 nm in diameter (Fig. 3b). Immunodeposition was also detected in the outer and inner loops of the myelinated axons, where fibril formation was not apparent (Fig. 3c).

### Distribution and incidence

The distribution and semiquantitative assessment of SCCIs in patients with MSA are summarized in Table 2. SCCIs were present in the cranial nerves (oculomotor, glossopharyngeal-vagus and hypoglossal nerves) and the spinal nerve roots. In the spinal nerve roots, SCCIs were found in the anterior nerves at the levels of the cervical, thoracic, lumbar and sacral segments, as well as in the posterior nerves in all the segments, except at the cervical level. They were also seen in the dorsal root and sympathetic ganglia and visceral autonomic nervous system.

**Table 2:** Distribution and frequency of Schwann cell cytoplasmic inclusions (SCCIs) in patients with multiple system atrophy

Case No.	Cranial nerves				Spinal nerves								DRG	SG	Visceral organs
					C		T		L		S				
	III	V	IX/X	XII	A	P	A	P	A	P	A	P			
1	NE	−	+	+	NE	NE	+	+	++	−	++	+	NE	NE	+ (stomach)
2	−	−	−	−	−	−	−	−	−	−	−	−	−	−	−
3	−	NE	+	−	+	−	−	−	+	−	+	+	+	−	−
4	−	−	+	NE	NE	NE	+	−	+	−	+	−	NE	NE	−
5	+	NE	−	−	−	−	+	−	+	−	+	−	−	+	−
6	NE	NE	+	−	+	−	−	−	−	−	+	−	+	−	−
7	NE	NE	−	−	+	−	−	−	−	−	+	−	+	−	−
8	++	NE	+	NE	−	−	−	−	−	−	−	+	+	−	−
9	NE	−	+	NE	+	−	+	+	−	−	+	+	NE	NE	−
10	NE	NE	−	−	+	−	+	−	−	−	+	−	+	−	−
11	−	NE	−	−	−	−	−	−	−	+	+	−	−	+	−
12	−	−	−	−	−	−	−	−	−	−	−	−	NE	NE	+ (adrenal, urinary bladder)
13	NE	NE	−	−	−	−	+	−	−	−	−	−	+	+	−
14	NE	−	NE	−	−	−	−	−	−	−	NE	NE	NE	NE	−
Percent positive for inclusions	28.6	0	46.2	9.1	41.7	0	42.9	14.3	28.6	7.1	69.2	30.8	66.7	33.3	14.3

DRG, dorsal root ganglia; SG, sympathetic ganglia; C, cervical; T, thoracic; L, lumbar; S, sacral; A, anterior; P, posterior. Semiquantitive assessment:−, none; +, 1 to 5 SCCIs per area; ++, more than 5 SCCIs per area; NE, not examined

SCCIs were found in 12 of 14 patients with MSA (85.7 %). They were most frequent in the anterior nerves of the sacral cord (69.2 %) and tended to be more frequent in the anterior than in the posterior nerves at each level. In one case of MSA (case 1), we examined the proximal and distal portions of the sacral nerve roots, and found that SCCIs were more numerous in the proximal than in the distal portion. In the cranial nerves, the inclusions were more frequent in the glossopharyngeal-vagus nerves (46.2 %) than in the oculomotor (28.6 %) and hypoglossal (9.1 %) nerves. SCCIs were found in 66.7 % and 33.3 % of the dorsal root and sympathetic ganglia, respectively. A small number of SCCIs were also found in the visceral organs in 2 of 14 patients with MSA (14.3 %): the subserosal nerves of the stomach in one patient (case 1) and the adrenal gland and urinary bladder in the other (case 12). There appeared to be no relationship between the frequency of SCCIs and the disease duration or clinical phenotype (MSA-C vs MSA-P) of patients with MSA.

Several neuronal cytoplasmic inclusions were found in the dorsal root ganglia in 2 of 9 MSA patients (cases 5 and 13) (Fig. 1 v, w). No such inclusions were found in the sympathetic ganglia or visceral organs.

## Discussion

In the present study, we have demonstrated for the first time that phosphorylated α-synuclein accumulates in the cytoplasm of Schwann cells in patients with MSA. These SCCIs were also immunopositive for aggregated α-synuclein, ubiquitin and p62, a ubiquitin- proteasome system-related protein. Thus, the immunohistochemical profile of SCCIs is similar to that of GCIs [3, 7, 9, 19, 25]. Ultrastructurally, SCCIs were composed of randomly arranged, loosely packed, granule-coated fibrils, approximately 15–20 nm in diameter. Both GCIs and neuronal cytoplasmic inclusions also consisted of granule-coated fibrils, approximately 20–30 nm in diameter [3, 4, 7, 26–28]. These findings indicate that Schwann cells are also involved in the disease process of MSA.

SCCIs were found in 12 of 14 patients with MSA (85.7 %) in the present study. GCIs were consistently found in the brainstem and spinal cord in all of the MSA patients. By contrast, SCCIs were not observed in the cranial or spinal nerves in three patients (cases 2, 12 and 14), two of whom had early MSA [17, 18]. These findings suggest that the occurrence of GCIs precedes that of SCCIs in MSA.

Recently, expression of human α-synuclein has been reported in Schwann cells ensheathing the nerve fibers of the urinary bladder in a transgenic mouse model of MSA showing oligodendroglial overexpression of human α-synuclein under the control of the proteolipid protein promoter [29]. Urodynamic analysis revealed a less efficient and unstable urinary bladder in this MSA mouse model. In human MSA, widespread occurrence of GCIs in the central nervous system is a cardinal pathological feature [3–6]. Moreover, neuronal cytoplasmic and nuclear inclusions have been observed in the inferior olivary and pontine nuclei, substantia nigra, putamen and cerebral cortex in patients with MSA [14, 28]. Filamentous aggregates of α-synuclein are also found in neurons in the sympathetic ganglia [14, 15]. In the present study, we further demonstrated that accumulation of phosphorylated α-synuclein occurs in the neuronal cytoplasm in the dorsal root ganglia. Sural nerve biopsy from patients with MSA shows a 23 % reduction of unmyelinated fibers (sensory afferent fibers and postganglionic sympathetic fibers) [30]. Mild degeneration of cardiac sympathetic nerves can occur in MSA [31]. Thus, MSA is a glio-neuronal α-synucleinopathy involving the central and peripheral nervous systems.

It is noteworthy that SCCIs tend to be more frequent in the peripheral nerves associated with autonomic function, i.e. glossopharyngeal-vagus nerves, and anterior spinal nerves of the thoracic and sacral cord. The vagus nerve is a mixed cranial nerve containing axons of branchiomeric motor neurons, parasympathetic preganglionic fibers, visceral afferent fibers, and somatic sensory afferent fibers. The glossopharyngeal nerve is related closely to the vagus nerve, sharing common medullary nuclei and having similar functional components [32]. The sympathetic ganglia receive preganglionic fibers from the intermediolateral nucleus of the spinal cord through the anterior roots of all the thoracic and the upper two lumber nerves [32]. The sacral preganglionic parasympathetic fibers exit from the sacral cord and go to the terminal ganglia of the pelvic plexuses, as well as to the myenteric and submucosal plexuses of the descending colon and rectum [32]. The widespread occurrence of SCCIs, at least in part, may play a role for the manifestation of a variety of autonomic symptoms in MSA.

Using the modified Gallyas-Braak method, GCIs were positive whereas SCCIs were stained only weakly or partially. Ultrastructurally, the constituent filaments of SCCIs (approximately 15–20 nm) appeared thinner than those of GCIs (approximately 20–30 nm) [3, 4, 7]. Phosphorylated α-synuclein-immunoreactive filamentous inclusions are also found in oligodendrocytes and astrocytes in the brains of patients with Parkinson’s disease and dementia with Lewy bodies [33–35] and are argyrophilic with the modified Gallyas-Braak method [36], suggesting that the process of α-synuclein aggregation in glial cells may differ somewhat between the central and peripheral nervous systems.

Cranial nerves are composed of myelinated and unmyelinated fibers in various proportions [37]. The nerve fibers of the anterior spinal nerve roots projecting to the autonomic ganglia are myelinated [38]. Both myelinated and unmyelinated fibers in the peripheral nervous system are enveloped with Schwann cells. Although the number of samples was small, our immunoelectron microscopy examination demonstrated that inclusion-bearing Schwann cells, at least in part, ensheath the myelinated fibers. Considering that postganglionic sympathetic nerve fibers are unmyelinated [39] and a small number of SCCIs were observed in the visceral autonomic nervous system in MSA, SCCI formation may also occur in Schwann cells ensheathing the unmyelinated fibers. Moreover, immunodeposition was also found in the outer and inner loops of Schwann cells. In the central nervous system, constituent filaments of GCIs are not evident in the outer or inner loops of oligodendrocytes in MSA [7]. By contrast, tau- and Gallyas-positive filamentous structures are found in the outer and inner loops of oligodendrocytes in progressive supranuclear palsy and corticobasal degeneration [39–41]. These findings suggest that phosphorylated α-synuclein pathology develops both in the perikarya and distal processes of Schwann cells, whereas the perikarya is chiefly involved in oligodendrocytes in MSA.

It is unclear how aggregated α-synuclein in the cytoplasm of Schwann cells interacts with the axon, myelin and Schwann cell itself. Both oligodendrocytes and Schwann cells are essential for axonal function and integrity. These enwrapping glia support axonal growth and myelination by transfer of metabolic substrates and secretion of neurotrophic factors [42]. Glial cell line-derived neurotrophic factor (GDNF) is one of the neurotrophic factors produced by oligodendrocytes [43] and Schwann cells [44]. The level of GDNF is significantly decreased in the frontal white matter and cerebellum of human MSA patients and in the brain of a MSA mouse model overexpressing human α-synuclein under the control of the myelin basic protein promoter [45]. Intraventricular infusion of GDNF improves behavioral deficits and ameliorates the neurodegenerative pathology in this MSA mouse model [45]. GDNF induces Schwann cell migration and axonal regeneration in the peripheral nervous system [46] and also prevents atrophy of facial motoneurons following axotomy [47]. Liver kinase B1 (LKB1) is also a crucial regulator of the major metabolic pathway in Schwann cells, which are central to axonal stability [48]. Deletion of LKB1 leads to energy depletion, mitochondrial dysfunction, abnormalities of lipid homeostasis and increased lactate release in Schwann cells [48]. The loss of viability in human neuroblastoma cells overexpressing wild-type α-synuclein is associated with reduced activation of intracellular energy sensors, including LKB1 [49]. α-Synuclein-overexpressing rat primary neurons also display lower LKB1 activity [49]. Based on the above findings, it is likely that overexpression of α-synuclein in Schwann cells impairs the activity of neurotrophic factors, leading to axonal destabilization in peripheral nerves.

The origin of α-synuclein in SCCIs is uncertain. Immunoreactivity of non-phosphorylated α-synuclein has been reported in normal and neoplastic Schwann cells in the peripheral nervous system of humans [16]. Therefore, it is possible to consider that overexpression of α-synuclein in Schwann cells would cause SCCI formation. As another possible mechanism, neuron-to-neuron transmission of α-synuclein fibrils through anterograde axonal transport has been demonstrated in primary cortical mouse neurons in vitro [50]. The fact that SCCIs tended to appear more frequently in the proximal than in the distal spinal nerve roots is appropriate for anterograde transport of α-synuclein. α-Synuclein in SCCIs could be derived from neurons. Future studies will be necessary to clarify the origin of α-synuclein in MSA Schwann cells.

## Conclusion

In conclusion, we have provided for the first time evidence that filamentous aggregation of phosphorylated α-synuclein occurs in Schwann cells in patients with MSA. Similar inclusions are also observed in the oligodendrocytes and neurons of the central nervous system as well as in neurons of the peripheral ganglia. Both central and peripheral mechanisms may contribute to the neurodegeneration in MSA.
